# STRENGTH IN SEEKING SUPPORT: OLDER LATINOS’ ATTITUDES ABOUT CANCER

**DOI:** 10.1093/geroni/igz038.2168

**Published:** 2019-11-08

**Authors:** Iraida V Carrion, Tania Estapé, Malinee Neelamegam, Jane Roberts, Jorge Estapé

**Affiliations:** 1 University of South Florida School of Social Work, Tampa, Florida, United States; 2 FEFOC, Fundacion Contra El Cáncer, Barcelona, Spain, Spain; 3 University of South Florida College of Public Health, Tampa, Florida, United States; 4 University of South Florida Sarasota-Manatee, Sarasota, Florida, United States

## Abstract

Given the growing Latino population 60 years and older, the current lack of relevant data, there is an urgent need to understand their attitudes about cancer to ensure effective prevention, intervention, and psycho-social care. A survey exploring attitudes about cancer was developed and administered in Spanish. Using convenience sampling (N = 168), univariate analysis was done to understand the study population’s characteristics. Frequencies were assessed to understand participants’ responses to questions on cancer-related attitudes. The effects of age, country of origin, length of stay in the U.S., and marital status were assessed using logistic regression. The participants had a mean age of 67.9 years, 65.5% were female, 56.5% were married or living with a partner, and 35.5% had tertiary education. Most respondents were from South America (46.7%), with a mean length of stay in the U.S. of 25.8 years. A high number (91.0%) indicated a preference to know if they had cancer, and 87.5% said that they would share their diagnosis with family and friends. Of the respondents, 80.4% felt that cancer patients should receive care from a psychologist and that cancer improves if a patient is lively and positive (82.6%). Compared to college-educated individuals, those with a high school education were less likely to choose to know about a cancer diagnosis (β=-1.92, p<0.01) or share it (β= 1.78, p<0.001). Attitudes about cancer vary depending on the educational level of older Latinos and may impact treatment decisions. These findings can enhance cancer information and education for older Latinos.

